# Simultaneous Transmission of Discrete-Variable Quantum Key Distribution and Classical Optical Communication in Few-Mode Fiber

**DOI:** 10.3390/e28030309

**Published:** 2026-03-09

**Authors:** Qi Zhao, Gang Wang, Li Pei, Jianjun Tang, Yuheng Xie, Zhenhua Li, Yang Liu

**Affiliations:** 1 China Telecom Research Institute, Beijing 102209, China; tangjj6@chinatelecom.cn (J.T.); xieyh@chinatelecom.cn (Y.X.); lizh84@chinatelecom.cn (Z.L.); liuyang19@chinatelecom.cn (Y.L.); 2 Group Cloud and Network Development Department, China Telecom, Beijing 100033, China; 3 Key Laboratory of All Optical Network and Advanced Telecommunication Network of Ministry of Education, Beijing Jiaotong University, Beijing 100044, China; lipei@bjtu.edu.cn

**Keywords:** quantum key distribution, few-mode fiber, coexistence system, spontaneous Raman scattering

## Abstract

Based on mode crosstalk theory, this paper develops a spontaneous Raman scattering (SpRS) model for the quantum-classical coexistence system using few-mode fiber (FMF) integrated with wavelength-division multiplexing (WDM) and spatial-division multiplexing (SDM). Through numerical calculations, the influence degrees of three factors (mode coupling, the number of modes and wavelengths) on SpRS are analyzed. The investigation identifies the dominant contributors to SpRS and reveals their relative impact magnitudes. Based on these results, a ring-assisted FMF is proposed to mitigate noise impacts on quantum signals. The numerical results show that the optimized FMF enhances quantum signal transmission distance by up to 41.5%.

## 1. Introduction

Quantum key distribution (QKD) generates secure keys between remote communication entities based on the fundamental principles of quantum mechanics, and it holds the potential to fundamentally transform the approach to securing information exchange in the future. In recent years, remarkable progress has been achieved in the research on QKD protocols and networks [1,2,3]. In 2023, the 1002 km twin-field QKD system broke the ultra-long fiber transmission limit for long-haul quantum communication, leveraging ultra-low-noise superconducting nanowire single-photon detectors (SNSPDs) and dual-band phase estimation [4]. Shortly afterward, multi-pixel SNSPDs combined with integrated transmitters increased short-haul QKD rates to over 110 Mbps while enhancing key efficiency [5]. In 2024, lightweight microsatellites and portable ground stations enabled real-time satellite-to-ground key distribution, advancing the development of global quantum networks. In 2025, 404 km entanglement-based QKD was successfully implemented [6].

However, the high cost of deploying dedicated fibers remains a major barrier to the large-scale application of QKD. A promising strategy to reduce deployment costs is the coexistence of QKD with classical optical communication. Coexistence systems based on wavelength-division multiplexing (WDM) or spatial-division multiplexing (SDM) technologies have been developed [7]. SDM technology, which exploits spatial degrees of freedom, is the primary direction for the future development of fiber optic networks. Initially proposed to address the capacity limitation of single-mode fiber (SMF) [8,9], various types of optical fibers have been developed for SDM implementation to date, including typical examples such as multi-core fiber (MCF), few-mode fiber (FMF), and few-mode multi-core fiber. Compared with MCF, FMF features a simpler fabrication process and can be directly spliced using conventional SMF fusion splicing equipment. Its large effective mode field area is conducive to mitigating nonlinear noise, making it well-suited for transmitting sensitive quantum signals [10].

Nevertheless, whether using MCF or FMF in coexistence systems, the SpRS generated by the classical signal is a challenge for this scheme. The wavelength range of SpRS exceeds 200 nm [11], which can easily affect the quantum channel and hinder the practical application of coexistence schemes. This study establishes an SpRS model for FMF-based coexistence systems and quantifies critical influencing factors. The ring-assisted FMF is proposed based on the analysis, achieving significant enhancement in quantum signal transmission distance.

## 2. Theoretical Model of SpRS When Quantum Signal Coexists with Classical Signal in FMF

In the coexistence system, each mode carries only classical or quantum signal. Different wavelengths are added to each mode using WDM. Since the proposed SpRS model builds on mode crosstalk, we first calculate the crosstalk mechanism as detailed in [12]. *P_i_* and *P_j_* denote the transmitted powers in each channel along the z-axis. Equations (1) and (2) can be obtained for the conditions that *P_j_*(0) = *p_c_* (classical channel initial power) and *P_i_*(0) = *p_q_* (quantum channel initial power) [13]. *α* is the attenuation coefficient in the channels, and *h*_ij_ is the power coupling coefficient.

(1) $$P_{j}(z)=\frac{1}{2}\left[\left(p_{c}+p_{q})\cdot \exp(-\alpha z)-(p_{c}-p_{q})\cdot \exp(-(\alpha +h_{ij})z\right)\right]$$

(2) $$P_{i}(z)=\frac{1}{2}\left[\left(p_{c}+p_{q})\cdot \exp(-\alpha z)+(p_{c}-p_{q})\cdot \exp(-(\alpha +h_{ij})z\right)\right]$$

Then, the derivation of SpRS is carried out, in which the classical signal of the *n*-th wavelength channel in *j*-th mode generates SpRS in the *m*-th quantum channel of the *i*-th mode. The SpRS arises through two distinct mechanisms: (1) direct crosstalk, where classical signals leak into quantum channel and directly generate SpRS; (2) induced crosstalk, where classical signals first produce SpRS in their original channels, and the generated SpRS subsequently crosstalks into the quantum channel.

In the first part, the power of SpRS can be described as [14]:

(3) $$dPL_{ICXT-SRS}(z)=\eta_{mn}P_{ICXT}(z)dz$$

where, *η*_mn_ denotes the Raman scattering coefficient between the *m*-th and *n*-th wavelength channels. The crosstalk power density at position *z*, *P_ICXT_*(*z*) is defined per differential length d*z* and can be calculated using Equation (2). To account for both SpRS generation and fiber attenuation effects, the SpRS power at the output end of the FMF is obtained by integrating contributions along the fiber length with appropriate loss:

(4) $$dP_{ICXT-SRS}(z)=dPL_{ICXT-SRS}(z)\exp[-\alpha_{q}(L-z)]$$

(5) $$P_{ICXT-SRS}=\int_{0}^{L}dP_{ICXT-SRS}(z)dz=\frac{1}{2}\eta_{mn}\cdot e^{-\alpha_{i}L}\cdot \left\{\frac{p_{c}+p_{q}}{\alpha_{i}-\alpha_{j}}[e^{(\alpha_{i}-\alpha_{j})L}-1]\right.\left.-\frac{p_{c}-p_{q}}{\alpha_{i}-\alpha_{j}-2h_{ij}}[e^{(\alpha_{i}-\alpha_{j}-2h_{ij})L}-1]\right\}$$

where *L* is the fiber length.

Next, we analyze the second contribution to SpRS. The SpRS power generated within a fiber segment d*z* in the *j*-th mode can be expressed as:

(6) $$dPL_{SRS-ICXT}(z)=\eta_{mn}P_{CS}(z)dz$$

*P*_cs_(*z*) represents the signal power at the differential segment *dz*, which can be calculated using Equation (1). As described in Equation (7), this power partially couples into the *i*-th mode and propagates to the output port of the FMF.

(7) $$dP_{SRS-ICXT}(z)=dPL_{SRS-ICXT}(z)\exp[-\alpha_{q}(L-z)]$$

The power equation can be derived as [15,16]

(8) $$P_{SRS-ICXT}=\int_{0}^{L}dP_{SRS-ICXT}(z)dz=\frac{1}{2}\eta_{mn}\cdot e^{-\alpha_{i}L}\cdot \left\{\frac{p_{c}+p_{q}}{\alpha_{i}-\alpha_{j}}[e^{(\alpha_{i}-\alpha_{j})L}-1]\right.\left.-\frac{p_{c}-p_{q}}{\alpha_{i}-\alpha_{j}-2h_{ij}}[e^{(\alpha_{i}-\alpha_{j}-2h_{ij})L}-1]\right\}$$

The total SpRS power in the system is determined by the derived equations:

(9) $$P_{SRS}=P_{ICXT-SRS}+P_{SRS-ICXT}=\eta_{mn}\cdot e^{-\alpha_{i}L}\cdot \left\{\frac{p_{c}+p_{q}}{\alpha_{i}-\alpha_{j}}[e^{(\alpha_{i}-\alpha_{j})L}-1]\right.\left.-\frac{p_{c}-p_{q}}{\alpha_{i}-\alpha_{j}-2h_{ij}}[e^{(\alpha_{i}-\alpha_{j}-2h_{ij})L}-1]\right\}$$

For the case of multiple classical channels in the system, the SpRS of the *m*-th channel in *i*-th mode (quantum channel) can be expressed as

(10) $$P_{SpRS}^{i,m}=\sum_{j=1}^{J}\sum_{n=1}^{N}P_{SRS}^{j,n}$$

According to the derivation results, the SpRS power in FMF is determined by fiber length, initial signal power, *α* and *h_ij_*. Among them, *h_ij_* is closely associated with the inherent properties of the fiber, which dominates the mode coupling in the FMF. Currently, research on *h_ij_* remains limited in coexistence systems utilizing other fibers. *h_ij_* can be expressed as [17]

(11) $$h_{ij}=\frac{2d_{c}{\left|\kappa_{ij}\right|}^{2}}{1+{\left[d_{c}(\beta_{i}-\beta_{j})\right]}^{2}}$$

**Figure 1:**
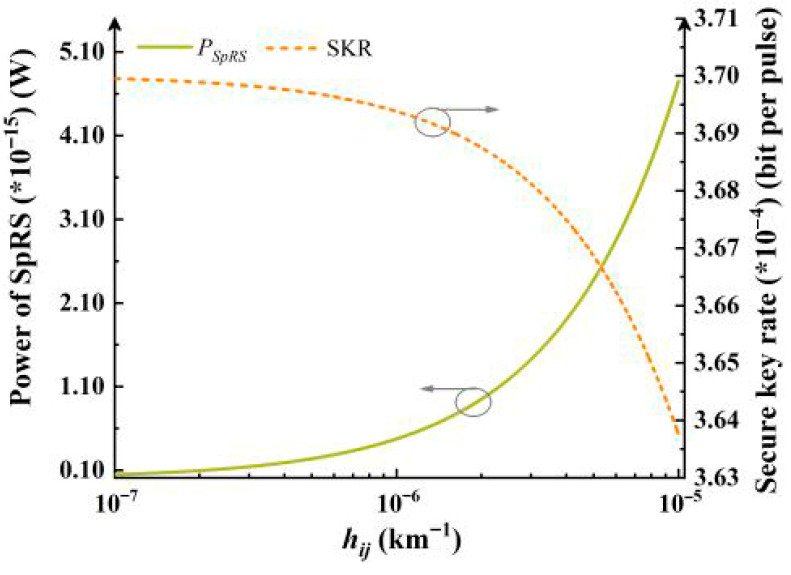
Power of SpRS versus *h_ij_* (green line), and SKR versus *h_ij_* (orange dashed line).

*β* is a propagation constant. *κ_ij_* is a mode coupling coefficient, which can be calculated as [18]

(12) $$\kappa_{ij}=\frac{k_{0}^{2}}{\beta_{j}}\iint \left(n^{2}-n_{0}^{2}\right)f_{i}^{*}(x,y)f_{j}(x,y)dxdy$$

where (*n*^2^ − *n*_0_^2^) is the refractive index perturbation, and *f*(*x,y*) is the mode field distribution. Figure 1 illustrates the relationship between SpRS, SKR and *h_ij_*. By modeling SpRS as equivalent noise photons, *p_SRS_* can be expressed as [19,20],

(13) $$p_{SRS}=\frac{P_{SpRS}\times \Delta f\times \Delta t}{h\times f}$$

*h* is the Planck’s constant. *f* is the frequency of the quantum channel. Δ*f* is the receiving bandwidth of the quantum channel, and Δ*t* is the detector effective gating width [13]. Taking the widely used BB84 protocol with the decoy-state method as an example, the SKR of a quantum signal can be described as [21]

(14) $$R=q\left\{-Q_{\mu }f_{e}H_{2}(E_{\mu })+Q_{1}[1-H_{2}(e_{1})]\right\}$$

*H_2_* is the binary Shannon entropy, *q* is set to 1/2 for the BB84 protocol, *f_e_* accounts for the efficiency of error correction which is set to 1.15. *μ* is the average number of photons in one pulse. *Q*_1_ and *e*_1_ are the gain and the error rate of the single-photon state. *Q_μ_* and *E_μ_* are the overall gain and the quantum bit error rate, respectively, which can be obtained by the noise count including the dark count of the single-photon detector and *p_SRS_*. According to Equation (14), the SKR is calculated. As *h_ij_* increases, the power of SpRS grows significantly, and the elevated noise subsequently degrades the SKR of the quantum signal, as shown in Figure 1.

## 3. Influencing Factors on SpRS in Coexistence System

**Figure 2:**
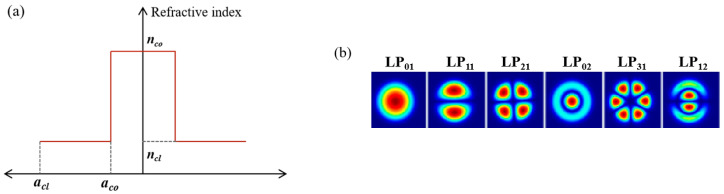
The radius and refractive index distribution of (**a**) step-index fibers and (**b**) the modes that can be supported in the fibers [13].

The previous analysis investigates the impact of mode coupling (*h_ij_*) induced by SDM technology on SpRS and quantum signal performance. Additionally, the variation in the number of wavelengths caused by WDM also affects the SpRS power. To further enhance the overall system performance, the subsequent research will analyze and compare the influence of classical signal wavelength and mode number in a coexistence system. The analysis focuses on common FMF, specifically four-mode (4-MF) and six-mode (6-MF) fibers. The fiber structures are illustrated in Figure 2a, while the supported modes for each fiber are detailed in Figure 2b (4-MF: LP_01_, LP_11_, LP_21_, and LP_02_; 6-MF: LP_01_, LP_11_, LP_21_, LP_02_, LP_31_, and LP_12_). The radius and refractive index are listed in Table 1. In the discussion, the quantum channel is assigned to the LP_01_ mode, while other modes serve as classical channels. Based on Equations (11) and (12), the *h_ij_* between LP_01_ and other modes can be derived, with results summarized in Table 2. The quantum channel operates at 1550.12 nm, while the classical signals employ ITU-standard channels with a channel spacing of 0.8 nm. The wavelength-dependent coefficient *η*_mn_ between quantum and classical channels is adopted from Ref. [20]. The launch powers of the quantum signal and classical signals are set to −80 dBm and 0 dBm, respectively. According to the data from YOFC, the attenuation coefficients α are set to 0.2 dB/km for LP_01_ and LP_11_, 0.21 dB/km for LP_21_ and LP_02_, and 0.215 dB/km for LP_31_ and LP_12_. Numerical analysis is performed based on the above parameters.

**Figure 3:**
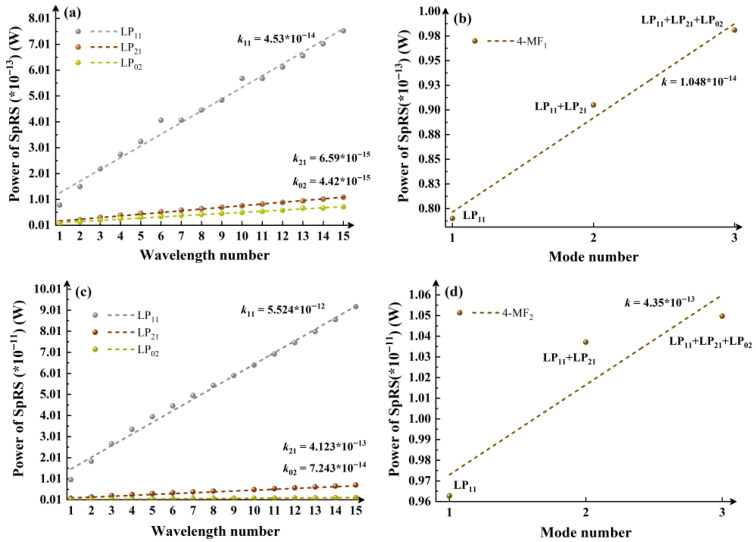
4-MF_1_: (**a**) Variation in SpRS power with an increasing number of wavelengths in the mode channel under single-mode classical signal. (**b**) Variation in SpRS power as the number of occupied modes by classical signals increases from 1 to 3 under a single wavelength. 4-MF_2_: (**c**) Variation in SpRS power with an increasing number of wavelengths in the mode channel under single-mode classical signal. (**d**) Variation in SpRS power as the number of employed modes by classical signals increases from 1 to 3 under a single wavelength.

Figure 3 shows a comparative analysis of the influence of wavelength and mode number on SpRS in 4-MF. Specifically, Figure 3a depicts the variation in SpRS power in the 4-MF_1_ fiber when classical signals employ single mode, as the number of wavelengths in the mode channel increases (from 1542.14 nm to 1554.13 nm, increasing by an interval of 0.8 nm according to the ITU standard, excluding 1550.12 nm). Figure 3b illustrates the evolution of the SpRS power in the 4-MF_1_ fiber when each mode channel contains only a fixed wavelength (1542.14 nm), while the number of modes occupied by the classical signal increases from 1 to 3 (successively activating the LP_11_, LP_21_ and LP_02_ mode). Similarly, Figure 3c,d correspond to the calculation results for the same scenarios in 4-MF_2_, where (c) represents the change in the number of wavelengths under a single mode and (d) represents the change in the number of modes under a single wavelength. The slopes of SpRS power increase under different conditions are listed in the figure to provide a direct comparison of the impacts from classical signal modes and wavelengths on SpRS. In Figure 3a,c, *k*_11_ is defined as the slope of SpRS power growth when the classical channel operates in LP_11_ mode, while *k*_21_ and *k*_02_ are assigned to LP_21_ and LP_02_ modes, respectively. From Figure 3a,c, a significantly accelerated SpRS power growth is observed when the classical channel uses LP_11_ mode, with *k*_11_ exceeding slopes of other modes by an order of magnitude (10^−14^ vs. 10^−15^). A comparative analysis of mode- and wavelength-dependent effects is performed through the calculated results in Figure 3a–d. It is observed that, excluding LP_01_ mode cases, SpRS growth trends induced by wavelength number increments under single-mode operation (Figure 3a,c) generally exhibit lower growth rates compared to those caused by mode number increments under fixed single-wavelength conditions (Figure 3b,d).

**Figure 4:**
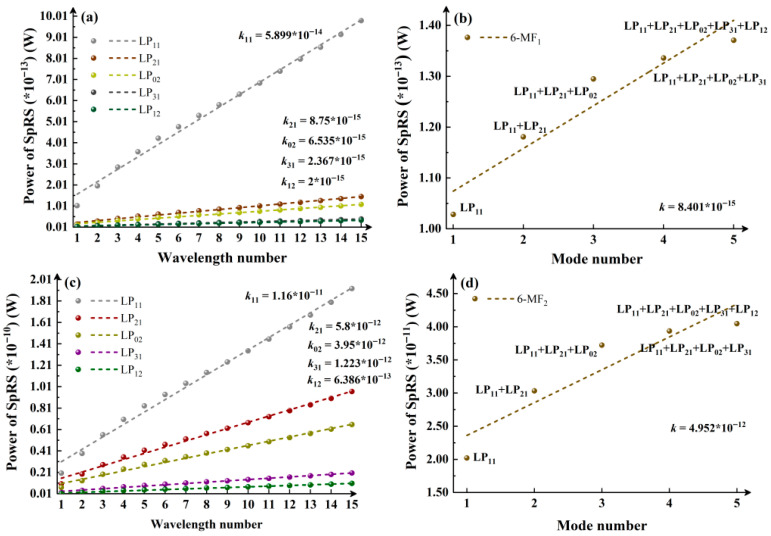
6-MF_1_: (**a**) Variation in SpRS power with an increasing number of wavelengths in the mode channel under single-mode classical signal. (**b**) Variation in SpRS power as the number of occupied modes by classical signals increases from 1 to 3 under a single wavelength. 6-MF_2_: (**c**) Variation in SpRS power with an increasing number of wavelengths in the mode channel under single-mode classical signal. (**d**) Variation in SpRS power as the number of employed modes by classical signals increases from 1 to 3 under a single wavelength.

Further calculations are performed in 6-MF, as shown in Figure 4. Similarly, from Figure 4a,c, the SpRS power growth remains fastest when the classical channel operates in LP_11_ mode, with its slope exceeding those of other modes by nearly an order of magnitude (10^−14^ vs. 10^−15^). Consistently, the mode- and wavelength-dependent effects are analyzed using the results in Figure 4a–d. It is found that, excluding LP_01_ mode cases, SpRS growth trends caused by increasing wavelength numbers under single-mode operation (Figure 4a,c) are generally lower than or comparable to those induced by increasing mode numbers under single-wavelength conditions (Figure 4b,d).

**Figure 5:**
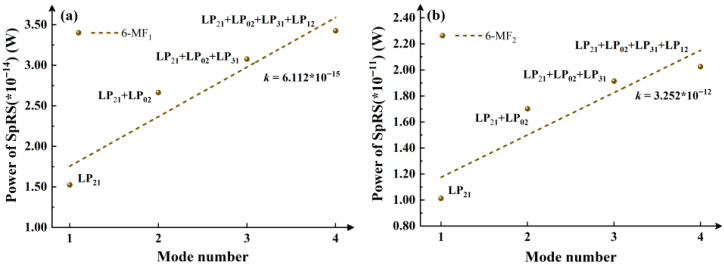
Under the condition of using 6-MF and excluding classical signal operating in the LP_11_, the growth trend of SpRS with the increase in the number of modes under the condition of a single wavelength. (**a**) 6-MF_1_ (**b**) 6-MF_2_.

Considering that the LP_11_ mode has a relatively large influence on SpRS, which may lead to errors in the data trend, the situation where the classical signal is in the LP_11_ mode is excluded in Figure 5. In the case of using a 6-MF, the growth trend of SpRS brought about by the increase in the number of modes when there is a single wavelength is analyzed. The activation order of the modes is LP_21_, LP_02_, LP_31_ and LP_12_. (The 4-MF only activates the two modes of LP_21_ and LP_02_, and it has no reference value.) By comparing the *k* values of the two groups of Figure 4a and Figure 5a, and Figure 4c and Figure 5b, it can be observed that under a single mode, the SpRS growth caused by wavelength increase remains lower than or comparable to the SpRS increase induced by mode number growth at a fixed wavelength. This result is consistent with the above discussion.

In summary, the analysis demonstrates that adjacent classical signal modes dominate quantum signal degradation compared to other factors. When adjacent modes exist, increasing the number of wavelengths generates more pronounced SpRS growth than adding modes. However, if the adjacent mode is removed, wavelength-induced SpRS growth becomes weaker or comparable to mode-induced growth. The quantified relationships derived from this analysis offer guidance for optimizing system performance.

## 4. Optimization and Performance Demonstration of FMF for Coexistence System

As demonstrated in Section 2 and Section 3, it can be seen that in addition to the number of modes and wavelengths, the mode coupling characteristics (*h*_ij_) of the FMF also have a great influence on the performance of the coexistence system. This section will focus on the optimization of *h*_ij_.

**Figure 6:**
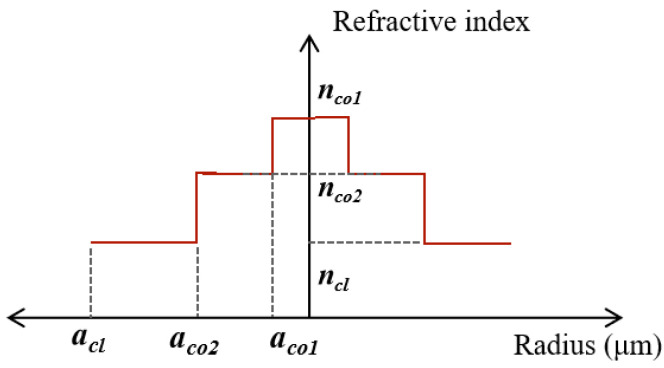
The radius and refractive index distribution of the ring-assisted FMF.

Owing to the high demultiplexing complexity of 6 MF in practical implementation, the optimization in this work is performed based on 4 MF. As demonstrated in previous studies, mode coupling is closely correlated with the effective refractive index difference Δ*n_eff_*. As indicated in Table 2 and Table 3, the mode coupling coefficient *h*_ij_ maintains a relatively low level under a large Δ*n_eff_*. Therefore, appropriately increasing Δ*n_eff_* is beneficial to suppressing the SpRS power and extending the transmission distance of quantum signals. The most direct approach to increasing Δ*n_eff_* is to enlarge the refractive index difference between the fiber core and cladding. However, for a fixed number of modes and given structural parameters, an overlarge Δ*n_eff_* will lead to a smaller mode field area, which is detrimental to the suppression of nonlinear effects [22]. To address this contradiction, a ring-assisted FMF is designed. By carefully selecting parameters of the refractive index ring in the core, the Δ*n_eff_* between each mode can be optimized. The optimized fiber structure is shown in Figure 6, with 4-MF_1_ as the basic design parameters. *a_co_*_1_ is the radius and *n_co_*_1_ is the refractive index of refractive index ring.

**Figure 7:**
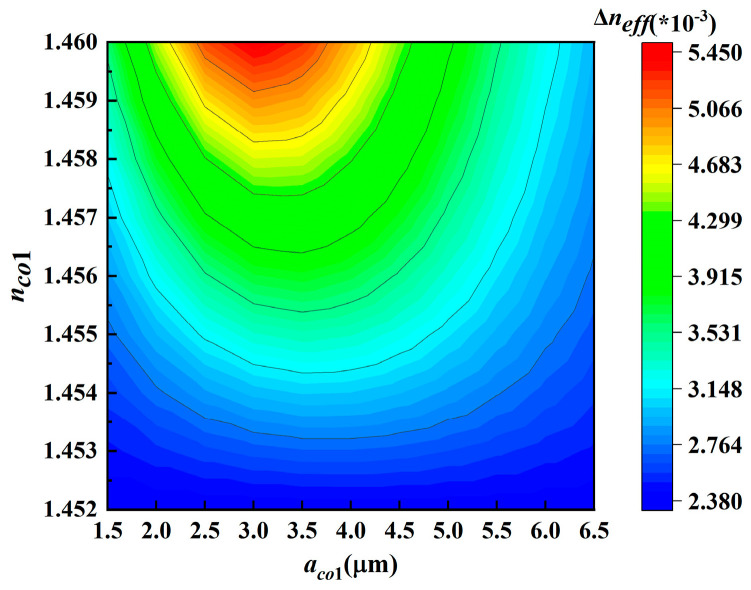
The variation in Δ*n_eff_* with *a_co1_* and *n_co1_*.

Considering the changes involving multiple parameters, the ergodic method is adopted for parameter design. The quantum signal is in the LP_01_ mode, and the LP_11_ mode has the greatest influence on it. Therefore, the primary optimization objective focuses on minimizing the Δ*n_eff_* between LP_01_ and LP_11_. Figure 7 illustrates the influence of variation in refractive index ring radius and refractive index on Δ*n_eff_*. The radius ranges from 1.5 nm to 6.5 nm with an interval of 0.5 nm, while the refractive index varies from 1.452 to 1.46 with an interval of 0.001. The distribution of Δ*n_eff_* can be intuitively observed in Figure 7, there are 21 parameter combinations exhibiting that the Δ*n_eff_* between LP_01_ and LP_11_ is greater than 4 × 10^−3^. Meanwhile, the Δ*n_eff_* between other modes should also strive to exceed 1 × 10^−3^.

Based on the influence of multiple parameters, the ring-assisted FMF parameters can be determined as *a_co_*_2_ = 7 μm, *a_co_*_1_ = 5 μm, *n_co_*_2_ = 1.452, *n_co_*_1_ = 1.46. Correspondingly, the Δ*n_eff_* and *h_ij_* of the ring-assisted FMF are summarized in Table 4. By comparing with the parameters listed in Table 2 and Table 3, it can be observed that the Δ*n_eff_* between LP_01_ and LP_11_ is increased, while the *h_ij_* values are significantly reduced.

**Figure 8:**
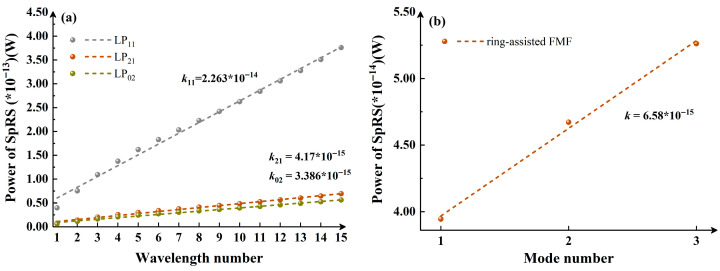
Theoretical verification: (**a**) variation in SpRS power with an increasing number of wavelengths in the mode channel under single-mode classical signal; (**b**) variation in SpRS power as the number of occupied modes by classical signals increases from 1 to 3 under a single wavelength.

**Figure 9:**
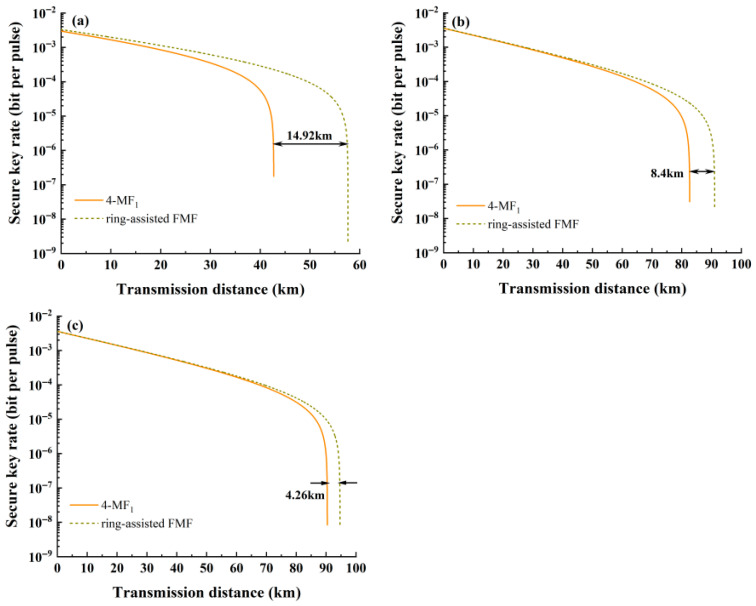
The relationship between SKR and transmission distance for classical signals in single mode: (**a**) LP_11_, (**b**) LP_21_ and (**c**) LP_02_.

The performance of the optimized ring-assisted FMF in the coexistence system is verified. Figure 8 further confirms the impacts of wavelength and mode number on the system. When the quantum channel operates in the LP_01_ mode and the classical channel is configured with a single mode (each mode is allocated 10 wavelengths via WDM), the variation in the SKR of key distribution with transmission distance is presented in Figure 9. Here, 4-MF_1_ is denoted by solid lines, while the ring-assisted FMF is indicated by dashed lines.

Evidently, the adoption of the ring-assisted FMF significantly enhances the transmission range of quantum signals. When the classical channel is configured in the LP_11_ mode, the transmission distance can extend up to 57.64 km, representing an impressive improvement of 34.9%. Meanwhile, when the classical channels are set to LP_21_ and LP_02_ modes, respectively, the corresponding transmission distances reach 91.05 km and 94.63 km, with respective improvements of 10.2% and 4.7%.

**Figure 10:**
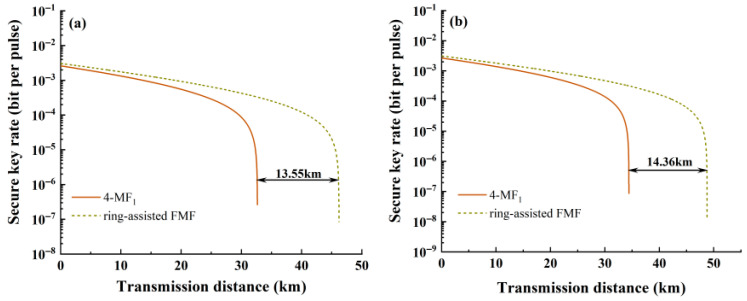
The relationship between SKR and transmission distance for classical signals under (**a**) three modes and (**b**) two modes.

A comparative analysis of the performance in multi-mode configurations is subsequently conducted. As shown in Figure 10, quantum signal transmission distances are compared between ring-assisted FMF and 4-MF_1_ fibers in Figure 10a, where LP_11_, LP_21_, and LP_02_ modes are simultaneously utilized as classical channels. Each mode is configured with 10 wavelengths spanning 1539.77~1546.92 nm with a interval of 0.8 nm. Figure 10b illustrates the transmission distance comparison for systems employing LP_11_ and LP_21_ modes under identical wavelength configurations. Significant improvements are observed with the ring-assisted FMF configuration: transmission distances are increased to 46.2 km (three modes) and 48.77 km (two modes), corresponding to enhancement rates of 41.5% and 41.7%, respectively. The results confirm the positive effect of optimization.

**Figure 11:**
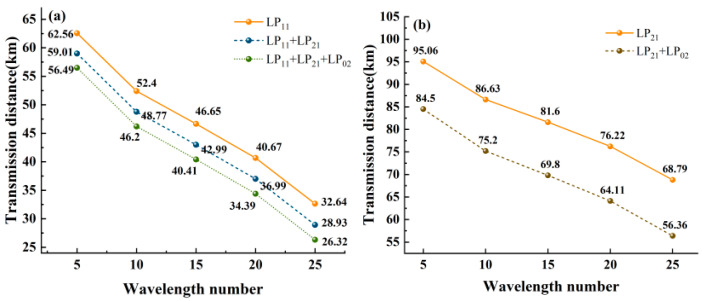
The variation in the transmission distance with the number of wavelengths of the ring-assisted FMF in different modes. (**a**) including LP_11_ mode (**b**) excluding LP_11_ mode.

Figure 11 presents the calculated transmission distances of quantum signals under different mode configurations and wavelength numbers. Figure 11a,b illustrate the transmission distances when utilizing 1~3 modes (including LP_11_ mode) and 1~2 modes (excluding LP_11_ mode), respectively. The results indicate that wavelength number exerts comparable or greater influence on transmission distance than mode number when employing LP_11_ mode, whereas mode number dominates in systems without LP_11_ mode. Moreover, the figures provide practical references for metropolitan area network applications by demonstrating the trade-off between transmission distance and channel capacity under different configurations. The number of wavelengths and modes can be selected according to the requirement.

**Figure 12:**
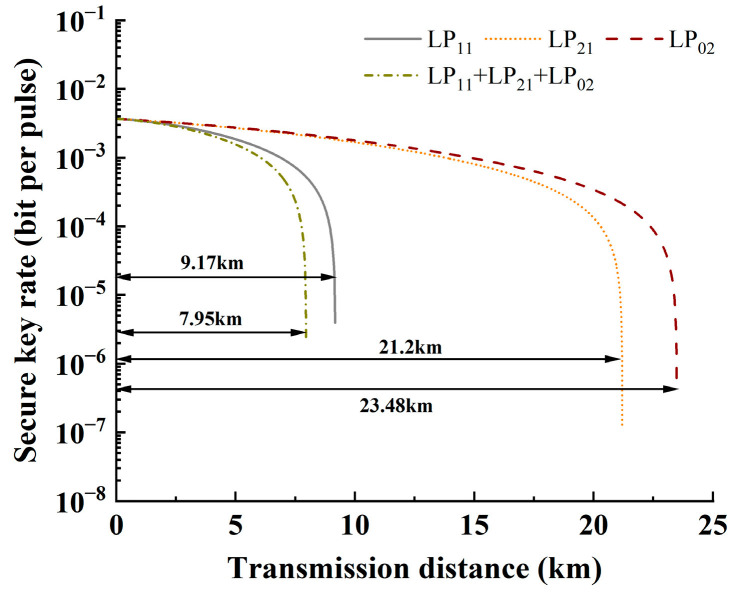
The relationship between SKR and transmission distance with 80 wavelengths in different modes.

In conventional optical communications, DWDM systems usually employ 80 wavelengths. Therefore, Figure 12 presents the variation in transmission distance when different modes are used for classical signal transmission, with each mode carrying 80 wavelengths. On this basis, the performance of the QKD system based on the optimized ring-assisted fiber is evaluated under extreme conditions, which can provide a reference for the practical application of such systems.

## 5. Conclusions

In this paper, an SpRS model based on FMF for the quantum-classical coexistence system is derived, and the factors affecting the SpRS within the system are analyzed and compared. To effectively mitigate the effects of SpRS, a ring-assisted FMF is devised to minimize power coupling between modes, thereby significantly enhancing the transmission distance of signals in system. In practical applications, the number of wavelengths and channel modes can be selected according to the requirements.

## Figures and Tables

**Table 1:** The radius and refractive index of each fiber.

	*n_co_*	*n_cl_*	*a_cl_* (μm)	*a_co_* (μm)
4-MF_1_	1.452	1.444	62.5	7
4-MF_2_	1.45	1.444	62.5	7.5
6-MF_1_	1.456	1.444	62.5	8
6-MF_2_	1.454	1.444	62.5	9

**Table 2:** The *h_ij_* of the FMF.

	LP_01_&LP_11_	LP_01_&LP_21_	LP_01_&LP_02_	LP_01_&LP_31_	LP_01_&LP_12_
4-MF_1_	3.72 × 10^−4^	5.40 × 10^−5^	3.55 × 10^−5^	-	-
4-MF_2_	6.7691 × 10^−2^	3.5761 × 10^−3^	5.9524 × 10^−4^	-	-
6-MF_1_	4.8449 × 10^−4^	7.1666 × 10^−5^	5.3517 × 10^−5^	1.9382 × 10^−5^	1.6384 × 10^−5^
6-MF_2_	9.7320 × 10^−1^	7.3291 × 10^−2^	4.1994 × 10^−2^	1.0743 × 10^−2^	5.4185 × 10^−3^

**Table 3:** The Δ*n_eff_* of the FMF.

	LP_01_&LP_11_	LP_11_&LP_21_	LP_21_&LP_02_	LP_02_&LP_31_	LP_31_&LP_12_
4-MF_1_	2.4	2.9	0.6	-	-
4-MF_2_	1.9	2.4	0.3	-	-
6-MF_1_	2.1	2.8	0.9	2.4	1.6
6-MF_2_	1.7	2.2	0.7	1.9	1.2

**Table 4:** The Δ*n_eff_* and *h_ij_* of the ring-assisted FMF.

	Δ*n_eff_* (*10^−3^)			*h_ij_* (km^−1^)		
	LP_01_&LP_11_	LP_11_&LP_21_	LP_21_&LP_02_	LP_01_&LP_11_	LP_01_&LP_21_	LP_01_&LP_02_
Ring-assisted FMF	4.0	4.7	1.0	1.8551 × 10^−4^	3.4145 × 10^−5^	2.7724 × 10^−5^

## Data Availability

Data are contained within the article.
